# Coordinating Care Aspects Related to Sexual Health in the Aging Male

**DOI:** 10.1155/2014/653587

**Published:** 2014-04-14

**Authors:** Antonio Aversa, Lorenzo Maria Donini, Roberto Bruzziches, Roberto LaCava, Francesco Mattace Raso, Alan Sinclair

**Affiliations:** ^1^Department of Experimental Medicine, Faculty of Medicine, Sapienza University of Rome, 00161 Rome, Italy; ^2^Department of Territorial Geriatric Medicine, ASP Catanzaro, 88100 Catanzaro, Italy; ^3^Department of Internal Medicine, Erasmus University Medical Center, 3015 CE Rotterdam, The Netherlands; ^4^Bedfordshire and Hertfordshire Postgraduate Medical School, Birmingham B29 6JD, UK

Global demographic changes demonstrate that the number of men aged 65 and older continues to increase dramatically. In order to attain a high level of health and well-being, important interventional areas such as diet, exercise, and sexuality may have important implications for older men.

Ageing males are at higher risk of several geriatric conditions, including falls from postural hypotension or balance and gait impairment, polypharmacy (more than three prescription medications), and use of sedative-hypnotic medications. These may be overshadowed by the development of prostate cancer, cardiovascular disease (CVD), depression, and obesity. It is known that obese men and women have a lower quality of life when compared with lean counterparts. In this issue, Poggiogalle et al. investigated the quality of life and more specifically the quality of sexual activity, in 95 obese men and women. Although the number of included patients was relatively small, the authors found an inverse association between body mass index and sexual activity in both men and women. In men, the results were mostly driven by erectile dysfunction and lack of sexual desire, whereas women reported a reduction of sexual disturbances with aging.

Aging men experience a gradual decline in serum testosterone (T), known as late onset hypogonadism (LOH), a condition that is often neglected by clinicians. It is estimated that hypogonadism affects between 19 and 40% of men over the age of 65. Consequently, understanding this phenomenon and its relationship with changes in body composition in older males is important because of associated conditions such as the metabolic syndrome, type 2 diabetes mellitus or obesity, CVD, and chronic heart failure. Hackett et al. give an update on the risk/benefit ratio of T supplementation on cardiac function in older men. They conclude that men with low T usually present with troublesome symptoms, particularly ED, and require treatment to address those problems, as well as addressing cardiovascular prevention. A considerable body of evidence suggests that low T is associated with increased CVD and cancer mortality. A policy of taking little or no action for these men based on concerns of increased cardiovascular and cancer risk associated with physiological replacement would seem illogical. There is considerable evidence of modest cardiac and metabolic benefits that are shown to reduce cardiovascular risk, plus sexual, mood, and quality of life changes associated with restoring T levels. These may also provide benefits to particular subgroups of frail older men thus avoiding fears over prostate and cardiac risk that must be supported by larger clinical studies in such populations.

The risk/benefit ratio of T replacement therapy on different organs, alone or in combination with nutraceuticals, is also a matter of debate in the aging male. In particular, together with the role of nutritional status as key factor of successful aging, mineral assessment has received attention as an important determinant of physical performance. There is evidence that magnesium exerts a positive influence on anabolic hormonal status, including testosterone, in men. Maggio et al. in their review summarize data from observational and intervention studies about the role of magnesium in testosterone bioactivity and the potential underlying mechanisms of this relationship in male subjects.

Gradual changes associated with male aging in the male reproductive system may include changes in testicular tissue, sperm production, and erectile function. Emerging data suggest that bone mass, energy metabolism, and reproduction may be coregulated by changes in body composition and visceral fat amount. It is now accepted that bone is an endocrine organ favouring whole-body glucose homeostasis and energy expenditure. These functions of bone are, in part, mediated by an osteoblast-specific secreted molecule, osteocalcin (OSCA). The potential relationship between circulating levels of OSCA and T with adipose tissue and bone mineral density in obese men has been evaluated by Migliaccio et al. in an original study carried out in an outpatient male clinic population. In their study, they propose for the first time the pivotal role of OSCA in the regulation of bone, metabolism, and reproductive functions.

Erectile dysfunction (ED) is a concern for many aging men. Erections occur less frequently as men age, and aging men often have less ability to experience repeated ejaculation. The underlying problem is likely to be due to medical reasons in about 90% of cases. Several medications (especially those used to treat hypertension and certain other conditions) may cause some men to be unable to develop or maintain enough of an erection for intercourse or to alter ejaculations. Gareri et al. presented an update in this area especially with regard to geriatric populations. Since data from pooled analyses are scarce, they found it difficult to recommend a specific optimal treatment for older men with comorbidities. However, they conclude that a Vardenafil orodispersible preparation is reported to have the fastest onset of action (pending the postmarketing data arriving soon from Avanafil, which is supposed to be as fast as ten minutes after assumption), while Tadalafil is reported to have the longer duration of action, with both representing a safe approach in an aged population. The novel longer-term use of phosphodiesterase type 5 inhibitors for other than ED conditions, that is, Sildenafil in pulmonary hypertension and Tadalafil in lower urinary tract symptoms (LUTS), appears to be promising ideas.

Benign prostate hyperplasia (BPH) may eventually interfere with urination and render older men more likely to have LUTS and/or prostatitis, especially in the presence of LOH. Pathogenic interconnections between BPH, inflammation, metabolic syndrome (MetS), and LOH are shown in the review by Corona et al. which highlights possible interventions to prevent their negative effect on men's health. BPH/LUTS represent a significant problem among aging men; they were historically considered as a normal consequence of the aging process and, as such, their negative effects on men's well-being were only dealt with through medical or surgical intervention. This view has been challenged in the last decade and now BPH/LUTS are seen more preventable than inexorable ailments of the older male population. Evidence presented in this review indicates that several modifiable metabolic factors play a role in determining the progression of LUTS/BPH. MetS and its components, hypogonadism and prostate inflammation, are, in fact, emerging as medical conditions commonly associated with BPH/LUTS, which in turn can be viewed as a complex disorder that involves a metabolic component that may begin early in the life of the male, remain asymptomatic, but is likely to be detectable even in the early stages of the disease. The mechanisms underpinning the relationship between MetS and prostate inflammation are likely to be similar in young and old men but chronic exposure to elevated inflammation, along with low T/high estradiol levels, may contribute to BPH in the long term. Preventing the development of the disease even from the asymptomatic phase should be the basis for designing a resilient program of elder healthcare.

Prostate cancer (PCa) becomes more common as men age. It is one of the most frequent causes of cancer death in men. Bladder cancer also becomes more common with ageing. All these situations may severely impair sexual behaviour and hence the quality of life of these individuals. Several trials on androgens and PCa have recently focused on urinary continence, quality of life, and sexual function, suggesting a new point of view on the whole endocrine aspect of PCa. In their review, Gacci et al. confirm that any treatment for PCa can have a long-lasting negative impact on quality of life and sexual health. In particular, sexual health, urinary continence, and bowel function can be worsened after prostatectomy, radiotherapy, or hormone treatment, mostly in the older population. The current knowledge on the role of hormones, metabolic aspects, and primary treatments for PCa in the quality of life and sexual health of older PCa survivors is analyzed in this review.

This issue has provided many insights into the management of older men with hypogonadism, especially as far as its related complications regard, that is, obesity, diabetes, and osteoporosis. Long-term T treatment of diabetic obese men with low T levels produced important clinical benefits in men with this combined syndrome, “diabesity.” Haider et al. collected and analyzed data from two observational, prospective, cumulative registry studies of 561 men with LOH receiving T therapy for up to 6 years. As far as we are aware, a unique aspect of this study is that it followed diabetic obese hypogonadal men for a period of 6 years, which is the longest reported duration of T treatment to date. Although the designs of the registry studies have some limitations, for example, they do not take into account behavioural and lifestyle changes, the authors conclude that T therapy of obese, diabetic men improves glycaemic control and lipid profiles and may prove useful in reducing the risk of CVD. In another original study, Haider et al. investigated the effects of normalizing serum testosterone on bone mineral density in 45 middle-aged to elderly men with osteoporosis, diagnosed with LOH. They demonstrated that T treatment not only improved their bone mineral density (from osteoporosis to osteopenia over six years of treatment), but also benefited their metabolic state, mood, and sexual functioning. Similarly, Francomano et al. show for the first time the results of a controlled, long-term study (60-month), on the effects of T replacement therapy in hypogonadal men with MetS. The authors confirmed the beneficial effects of a long-acting T injection on anthropometric and metabolic parameters defined as a reduction of cardiovascular risk factors (lipid profile, blood pressure, insulin resistance, and HBA1c). Noteworthy, a significant improvement in bone mineral density in relation to a concomitant increase in the levels of serum vitamin D was firstly reported, which is explained by a possible direct effect of testosterone on renal expression of the l-alpha-hydroxylase and on the reduction of fat mass independently from estradiol modifications. In addition, the add-on effects of T injections on circulating levels of pituitary hormones lead the authors to consider obesity as a “panhypopituitarism” condition determining a multiendocrine dysfunction. Last, but not least, the remarkable safety profiles of hormone replacement therapy on haematological and prostatic parameters were confirmed in all these studies.

As men approach older age with all their exuberance, they exit with physiological maturity. Let us just stop for a moment to meditate what are the physical and psychological impacts of such changes and what new complex mechanisms are going to change over the continuation of life. This is not the end of life itself, but it is a more complex interplay between maturity and wisdom. If an older man does not apply “*Virtute e Canoscenza*” he might be transformed into its opposite, that is, bewilderment and melancholy. This transmutation is the basis for the “alchemical” endocrinologist, who uses his tools to process the spirit and the senses. This issue provides new considerable progress in several important metabolic areas related to male sexual function and how this has such an important bearing on male health and well-being and the development of premature CVD in the aging male. We are beginning to be more objective about the benefits and risks of T therapy, seeing the complex relationship between MetS, obesity, and LUTS becoming unravelled, and gaining new insights into metabolic bone disease. This is an exciting time and at this current rate of enquiry this should prompt a further review in this area in three years!

*Antonio Aversa* *Antonio Aversa* *Lorenzo Maria Donini* *Lorenzo Maria Donini* *Roberto Bruzziches* *Roberto Bruzziches* *Roberto LaCava* *Roberto LaCava* *Francesco Mattace Raso* *Francesco Mattace Raso* *Alan Sinclair* *Alan Sinclair*

